# Post-randomization Differences in Condomless Vaginal Sex Among Women Randomized to Intramuscular Depot Medroxyprogesterone Acetate Injections, a Copper Intrauterine Device or a Levonorgestrel Implant in the ECHO Trial

**DOI:** 10.1007/s10461-022-03834-y

**Published:** 2022-11-11

**Authors:** Jennifer Deese, Pai Lien Chen, Xiaoming Gao, Renee Heffron, Marcia Hobbs, Dana Lapple, Heather Jaspan, Ashley Miller, Gonasagrie Nair, Maricianah Onono, Thesla Palanee-Phillips, Krishnaveni Reddy, Markus J. Steiner

**Affiliations:** 1 Present Address: Women’s Global Health Imperative, Global Public Health Impact Center, RTI International, Research Triangle Park, USA; 2 Biostatistics and Data Science, FHI 360, Durham, USA; 3 Department of Global Health and Department of Epidemiology, University of Washington, Seattle, WA USA; 4 University of North Carolina at Chapel Hill, Chapel Hill, NC USA; 5 Seattle Children’s Research Institute, Seattle, WA USA; 6 Science Facilitation, FHI 360, Durham, NC USA; 7 Centre for Medical Ethics and Law, Department of Medicine, University of Stellenbosch, Stellenbosch, South Africa; 8 Kenya Medical Research Institute, Nairobi, Kenya; 9 Faculty of Health Sciences, Wits Reproductive Health and HIV Institute, University of the Witwatersrand, Johannesburg, South Africa; 10 Product Development and Introduction, FHI 360, Durham, USA; 11 Cary, USA; 12 FHI 360, Durham, USA

**Keywords:** Prostate specific antigen, Sexual behaviour, Contraception

## Abstract

The Evidence for Contraceptive Options and HIV Outcomes (ECHO) trial found no substantial difference in HIV acquisition risk between women randomised to injectable intramuscular depot medroxyprogesterone acetate (DMPA-IM), copper intrauterine device (Cu-IUD) or the levonorgestrel (LNG) implant. We evaluated post-randomization sexual behavior using an objective marker of condomless vaginal sex in a subset of participants. We conducted a sub-study among 458 ECHO participants at three sites (Cape Town, Johannesburg, Kisumu) to evaluate the frequency of condomless vaginal sex, measured by prostate specific antigen (PSA) detection in vaginal swabs, collected at the month 6 and final visit and the concordance of self-reported condomless vaginal sex with PSA detection, by randomized arm. We compared PSA detection frequency and concordance of PSA and self-reported condomless vaginal sex, by randomized group using Cochran–Mantel–Haenszel tests and adjusted generalized logistic growth curve models. PSA was detected less frequently in the DMPA-IM (16%), compared to the Cu-IUD (21%) and LNG implant (24%) groups, although results were not statistically significant in the unadjusted model when accounting for pre-specified multiple-testing criteria. There were significant differences in PSA detection between the DMPA-IM and LNG-implant groups (odds ratio 0.61 (95% CI 0.40, 0.94) in the adjusted model. There was moderate discordance between self-reported condomless vaginal sex and detection of PSA that was similar across randomized groups. These data suggest that women randomized to Cu-IUD and LNG implant may have had condomless sex more frequently than women randomized to DMPA-IM. The discordance between detectable PSA and self-reported sexual behaviour has important implications for design of future HIV prevention studies.

## Introduction

The Evidence for Contraceptive Options and HIV Outcomes (ECHO) trial, which randomized women to three different contraceptive methods including intramuscular injectable depot medroxyprogesterone acetate (DMPA-IM), a copper intrauterine device (Cu-IUD) and a levonorgestrel (LNG) implant, found no significant differences in HIV acquisition between the contraceptive methods. Due to the nature of the contraceptive methods under study, neither study participants nor research staff at the study sites were blinded. We aimed to evaluate whether there were any post-randomization differences in condomless vaginal sex (hereafter sexual behavior), and thus potentially to HIV exposure, by randomized contraceptive group using prostate specific antigen (PSA) as an objective marker of recent condomless vaginal sex. PSA has been used in multiple studies as a valid proxy measure for recent condomless vaginal sex and is detectable in vaginal fluid immediately following condomless vaginal sex, and generally returns to undetectable levels within 48 h [1, 2]. We also compared detection of PSA to participant self-reported sexual behavior data to understand the accuracy of self-reported data in this context.

## Methods

### Study Design, Participants and Ethics

The ECHO Trial (clinicaltrials.gov ID NCT02550067) randomized 7829 HIV-seronegative women to either DMPA-IM, Cu-IUD or LNG implant, from December 2015 through September 2017 [3]. We conducted a prospective sub-study among ECHO trial participants to evaluate the frequency of condomless vaginal sex, as measured by detection of PSA in vaginal swabs, and the correlation of self-reported sexual behaviour with PSA detection, by randomized arm. The participants included in these analyses were those who consented to vaginal sample collection through an ancillary biological mechanisms study nested within ECHO to understand the impact of the study contraceptive methods on biological mechanisms of HIV acquisition. This sub study was conducted at three of the twelve ECHO trial sites including the Desmond Tutu HIV Centre, Cape Town and Wits Reproductive Health and HIV Institute, Johannesburg, South Africa and Kenya Medical Research Institute, Kisumu, Kenya [4]. To be eligible, participants had to have: (1) complete ECHO trial demographic (baseline; for identification of participant age) and self-reported sexual behaviour data (month six and final study visit conducted 12–18 months after enrolment) [3], (2) valid baseline and final visit nucleic acid amplification test (NAAT) results for *Chlamydia trachomatis* and *Neisseria gonorrhoeae*, (3) used their randomized contraceptive method for their duration of trial participation, and (4) stored vaginal specimens at the month six and final study visits.

This research was implemented in accordance with the Declaration of Helsinki and Good Clinical Practice. Institutional review boards at FHI 360, the University of Washington and each clinical site approved the study protocol and women provided written informed consent for future research on their biological samples.

### Specimen Collection and Analysis

Clinical staff collected a lateral vaginal wall swab from participants at each follow-up visit; swabs were stored dry at − 80 °C until analysis. We analysed swabs collected at the month six and final study visits for PSA at the University of North Carolina at Chapel Hill (UNC). Trained laboratory staff eluted lateral vaginal wall swabs into phosphate-buffered saline and centrifuged suspensions to separate cellular material and soluble contents (including PSA) in the supernatant using Isohelix Spin + Collect SC150-SPIN Devices. Eluate was diluted 1:10 with PBS and tested for PSA using the ABAcard p30 rapid immunochromatographic strip test (Abacus Diagnostics, West Hill, CA; lower limit of detection 4 ng PSA/ml) as previously described [1].

### Statistical Analysis

Under the assumption of an average 20% PSA positivity rate at each follow-up time point, and accounting for 20% correlation between repeated measures and type 1 error rate adjustment due to multiple comparisons between randomized groups, our a priori assessment suggested that a sample size of 450 (150 per group) would provide > 90% power to detect a 15% difference in PSA detection between randomized groups. To investigate differences in the frequency of condomless vaginal sex between randomized contraceptive groups, we calculated the frequency of PSA detection at the month six and final study visits. We then compared the proportion of PSA positive specimens by randomized contraceptive group at the month six and final visits separately using the Cochran–Mantel–Haenszel test stratified by study site with Bonferroni correction. We used a generalized logistic growth curve model adjusting for site, age (continuous) and specimen collection order to evaluate whether the proportion of PSA positive specimens differed significantly by randomized group and/or over time. We evaluated the concordance of self-reported sexual behaviour data and PSA detection in two ways by randomized study group: (1) the proportion of women who reported no vaginal sex, or only condom-protected vaginal sex in the past seven days, who had detectable PSA, and (2) the proportion of women who reported their last vaginal sex act to have been within 3 days with a condom, or reported their last vaginal sex act to have been ≥ 4 days ago whether or not they report condom use during that last act, who had detectable PSA. Using these data, we estimated the proportion of potential misreporting, and associated 95% confidence interval, overall and by randomized contraceptive group at the month 6 and final visits individually and jointly. The same statistical analysis approaches described above were used to compare potential misreporting by randomized contraceptive method and to evaluate whether the proportion of potential misreporting differed by group and over time. All data analyses were conducted using SAS version 9.4 (Cary, NC, USA).

### Participant and Public Involvement

The ECHO trial was designed over several years through an extensive consultative process with a range of stakeholders that included women’s health advocates. The trial’s Global Community Advisory Group (GCAG), which included advocates for women’s health and rights from each of the countries where the study was conducted, advised the ECHO Consortium on the trial protocol. The conduct of the trial was monitored through site-specific Good Participatory Practice plans designed to operationalize the GPP principles of respect, transparency, accountability, and community stakeholder autonomy.

## Results

### Participant Characteristics

A total of 558 randomized participants had stored month 6 and final visit samples available for analysis at the three participating sites. Among those, 458 met the aforementioned inclusion criteria and were included in this analysis, including 145 in the DMPA-IM group, 158 in the Cu-IUD group and 155 in the LNG implant group. Baseline characteristics were similar across groups (Table 1). The median age of participants was 23 years, about half (50.9%) were married and had completed any secondary school (54.6%). Participants reported a median of 10 vaginal sex acts per month in the three months prior to enrolment, the vast majority (96.9%) reported having had the same primary sex partner during that period and most (73.3%) women reported having had any unprotected sex. The prevalences of *Chlamydia trachomatis* and *Neisseria gonorrhoeae* were 15.5% and 4.1% respectively at baseline, with no significant differences by randomized group. Analysis of these baseline demographic, sexual behavior and STI prevalence data found no statistically significant differences between this sub-sample of ECHO trial participants and the overall trial population (data not shown).

**Table 1:** Participant baseline characteristics by randomized contraceptive group

	IUD N = 158 n (%)	DMPA N = 145 n (%)	Implant N = 155 n (%)	Total N = 458 n (%)	p-value^1^
Age (year)					
Median (Range)	23 (16 to 35)	23 (16 to 35)	22 (16 to 35)	23 (16 to 35)	0.259
Current marital status					
Never married	81 (51.3)	64 (44.1)	74 (47.7)	219 (47.8)	0.562
Married	77 (48.7)	78 (53.8)	78 (50.3)	233 (50.9)	
Previously married	0 (0.0)	3 (2.1)	3 (1.9)	6 (1.3)	
Total	158	145	155	458	
Participant living with husband/partner					
Yes	84 (53.2)	82 (56.6)	84 (54.2)	250 (54.6)	0.704
No	73 (46.2)	60 (41.4)	68 (43.9)	201 (43.9)	
N/A, no partner	1 (0.6)	3 (2.1)	3 (1.9)	7 (1.5)	
Highest level of education					
None	2 (1.3)	0 (0.0)	0 (0.0)	2 (0.4)	0.066
Any primary school	55 (34.8)	43 (29.7)	59 (38.1)	157 (34.3)	
Any secondary school	77 (48.7)	86 (59.3)	87 (56.1)	250 (54.6)	
Post-secondary school	24 (15.2)	16 (11.0)	9 (5.8)	49 (10.7)	
Number of vaginal sex acts past 3 months					
Median (interquartile range)	10 (3 to 20)	10 (3 to 20)	9 (3 to 20)	10 (3 to 20)	0.973
No condom use last vaginal sex act	76 (53.9)	65 (50.8)	70 (51.9)	211 (52.2)	0.872
*Chlamydia trachomatis*	32 (20.3)	18 (12.4)	21 (13.5)	71 (15.5)	0.121
*Neisseria gonorrhoeae*	8 (5.1)	7 (4.8)	4 (2.6)	19 (4.1)	0.482

^1^Kruskal-Wallis test (continuous variables) and exact Pearson chi-square test (categorical variables) are used to assess differences between randomized groups

### PSA Detection During Follow-Up

**Fig. 1:**
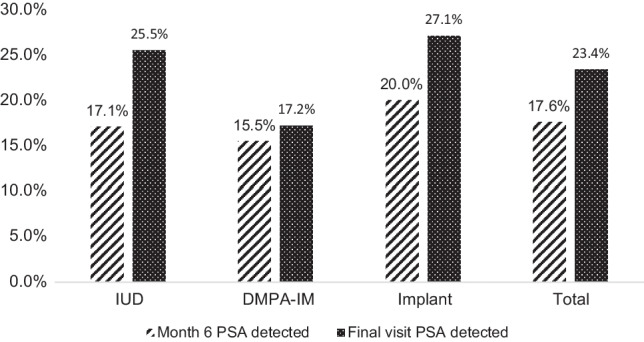
Proportion of samples with PSA detected at month 6 and the final visit

PSA was detected in 17.6% of month 6 and 23.4% of final visit vaginal swab specimens (Fig. 1). In general, women in the DMPA-IM group were less likely to have PSA detected at both the month 6 and final visits (15.5% and 17.2%) as compared to women in the Cu-IUD (17.1% and 25.5%) or LNG implant groups (20.0% and 27.1%, Table 2). When pooling across visits, women in the DMPA-IM group had 29% lower odds [odd ratio (OR) 0.71, 95% CI 0.46, 1.06)] of PSA detection compared to the copper IUD group and 39% lower odds [OR 0.61 (0.40, 0.94) compared to the LNG implant group. Women in the Cu-IUD arm had 13% lower odds of PSA detection than women in the LNG implant group (OR 0.87 (0.59, 1.31)]. Across all arms, women were significantly more likely to have PSA detected at the final visit relative to the month 6 visit [OR 1.44 (1.05, 1.98)].

**Table 2:** Pairwise comparison of PSA detection frequency by randomized contraceptive arm

Visit	Test of association	DMPA-IM vs. Cu-IUD	DMPA-IM vs. implant	Cu-IUD vs. implant
Month 6	CMH p-value^1^	0.708	0.301	0.487
Final	CMH p-value^1^	0.048	0.025	0.757
All visits	Logistic model OR (95% CI)^2^	0.70 (0.46, 1.06)	0.61 (0.40, 0.94)	0.87 (0.59, 1.31)
	Logistic model p-value^2^	0.095	0.024	0.512

^1^Cochran-Mantel–Haenszel test to compare proportion of PSA positive specimens pairwise between randomized contraceptive groups stratified by study site. A p-value < 0.0167 (0.05/3) is considered significant with Bonferroni correction

^2^Odds ratios from a logistic generalized estimating equation model adjusted for site, age and PSA collection order

### Correlation of PSA Detection and Self-reported Sexual Behavior

There was moderate discordance between self-reported sexual behavior and PSA results (Table 3). Of the women with detectable PSA at month 6 indicating condomless sex in the past 7 days (N = 80), 19% reported having had no vaginal sex, or only vaginal sex with a condom, in the past 7 days. Furthermore, 7.8% percent of women with detectable PSA at month 6 reported no vaginal sex in the past 7 days. Slightly higher discordance was observed at the final visit with 20 (25%) of the women with detectable PSA reporting no vaginal sex, or only vaginal sex with a condom, in the past 7 days. Sixteen percent of women with detectable PSA reported no vaginal sex in the past 7 days. There were no significant differences in discordance between randomized contraceptive groups [DMPA vs. Cu IUD OR 1.27 (95% CI 0.48, 3.34); DMPA vs. implant OR 1.72 (0.61, 4.86); IUD vs. implant OR 1.36 (95% CI 0.52, 3.55)].

**Table 3:** Discordance between PSA detection and self-reported sexual behavior among women with detectable PSA

Visit	Self-reported measure	Cu-IUD n (%)	DMPA-IM n (%)	Implant n (%)	Total n (%)
		N = 27*	N = 22*	N = 31*	N = 80*
Month 6	No vaginal sex past 7 days	3 (13.0)	0 (0.0)	2 (8.0)	5 (7.8)
	No vaginal sex or only condom-protected vaginal sex last 7 days	6 (26.1)	3 (18.8)	3 (12.5)	12 (19.0)

Visit	Self-reported measure	Cu-IUD n (%)	DMPA-IM n (%)	Implant n (%)	Total n (%)
		N = 40*	N = 25*	N = 42*	N = 107*
Final	No vaginal sex past 7 days	3 (10.3)	6 (30.0)	4 (12.9)	13 (16.3)
	No vaginal sex or only condom-protected vaginal sex last 7 days	6 (20.7)	7 (35.0)	7 (22.6)	20 (25.0)

*Number of specimens with PSA detected

## Discussion

These data suggest that the frequency of condomless sex may have differed by randomized contraceptive method among ECHO trial participants at the 3 (out of 12 total) ECHO sites in this sub-study. By objectively measuring condomless sex, these data confirm the prior suggestion of differences in by-arm sexual behavior that were generated from self-reported data [3]. Any true differences in sexual behavior by randomized contraceptive group could be due to side effects of the contraceptives, differences in vaginal practices and/or differential concerns about HIV risk. The contraceptive methods under study in ECHO have side effect profiles that could differentially affect women’s sexual behavior. DMPA-IM is associated with irregular bleeding and reduced libido which could result in decreased sexual frequency and thereby explain the lower PSA detection in that group [5]. However, both the Cu-IUD and LNG implant are also associated with bleeding irregularities, including amenorrhea, irregular bleeding, heavy bleeding and prolonged bleeding [6, 7]. The bleeding pattern irregularities with these methods tend to differ over time, thus making it difficult to draw conclusions about the impact of bleeding differences on sexual behavior by contraceptive arm. It is also possible that women in the DMPA-IM arm engaged in less frequent sex and/or more condom use due to concerns about HIV risk. While the informed consent process described that the study objective was to understand if *any* of the three contraceptive methods change a woman’s risk of HIV, it also explained that the World Health Organization stated (at the time) that there was evidence of a possible increased risk of HIV acquisition associated with DMPA-IM. It is therefore plausible that women in the DMPA-IM arm may have modified their sexual behavior due to concerns about HIV risk.

There are important limitations to this analysis that must be considered in interpretation of the results. While we did not observe any statistically significant differences in baseline demographic, sexual behavior or STI prevalence by randomized contraceptive group between the women in this sub-study as compared to the overall trial population, this sub-study represents only 6% of the total trial population. Moreover, the selection criteria for inclusion in this sub-study required that women have remained on their randomized contraceptive method through their final visit. It is possible that women in this sub-study differed in unknown ways correlated with sexual behavior that are not generalizable to the full trial population or the larger population of women represented by trial participants.

Importantly, the data from this study highlight reliability concerns with self-reported sexual behavior. While PSA is not a perfect surrogate for recent condomless vaginal sex due to inter- and intra-person variability PSA clearance kinetics, the self-reported measures we selected for comparison with PSA detection were robust. Specifically, between 8 and 16% of women with PSA detected reported not having any vaginal sex in the prior 7 days and PSA kinetic data clearly exclude detection of PSA at that timepoint [2]. The interpretation of PSA detection among women who reported no, or condom-only protected sex, in the last 7 days is more subject to error since women were not specifically asked about vaginal sex acts in which a condom was used *for the entire duration* of vaginal sex. Nonetheless, the data suggest that studies which rely on self-reported sexual behavior should consider validation against reliable biological indicators of recent condomless vaginal sex.

In conclusion, the results of this sub-study suggest that the frequency of condomless vaginal sex may have differed between randomized contraceptive groups in the ECHO trial. Notably, the ECHO trial was designed to evaluate whether there are differences in the risk of HIV acquisition among women randomized to DMPA-IM, the Cu-IUD and the LNG implant, and the trial accomplished that objective. While the trial was not designed to evaluate the biological effect of the randomized contraceptive methods on HV risk, the results from a causal analysis which adjusted for self-reported sexual behaviour generated results consistent with the intention-to-treat results [3]. The results of this sub-study suggest that inaccuracies in participant self-reported sexual behavior could have resulted in residual confounding of the causal analysis.

## Data Availability

Access to data from this ancillary study of the ECHO Study may be requested through submission of a research concept to: jdeese@rti.org. Access will be granted if the concept is evaluated to have scientific merit and if sufficient data protections are in place. As of the time of publication, data access applications are in process with the governing institutional review boards of the ECHO Study to make de-identified data from the full trial publicly available.

## Code Availability (Software Application or Custom Code)

Access to code from this ancillary study of the ECHO Study may be requested through submission of a request: jdeese@rti.org.
